# Meta-analysis of oxaliplatin-based versus fluorouracil-based neoadjuvant chemoradiotherapy and adjuvant chemotherapy for locally advanced rectal cancer

**DOI:** 10.18632/oncotarget.16127

**Published:** 2017-03-11

**Authors:** Xing-Li Fu, Zheng Fang, Liang-Hui Shu, Guo-Qing Tao, Jian-Qiang Wang, Zhi-Lian Rui, Yong-Jie Zhang, Zhi-Qiang Tian

**Affiliations:** ^1^ Department of General Surgery, Wuxi People's Hospital Affiliated Nanjing Medical University, Wuxi 214023, China; ^2^ Department of Biliary Surgery, The Eastern Hepatobiliary Surgery Hospital, Second Military Medical University, Shanghai 200438, China; ^3^ Health Science Center, Jiangsu University, Zhenjiang, Jiangsu 212001, China; ^4^ Department of Nephrology and Endocrinology, The 101st Hospital of Chinese PLA (Wuxi Taihu Hospital), Wuxi 214044, China; ^5^ The Second People's Hospital of Jintan District, Changzhou, Jiangsu 213200, China; ^6^ The People's Hospital of Liyang, Changzhou, Jiangsu 213300, China

**Keywords:** rectal cancer, neoadjuvant chemoradiotherapy, adjuvant chemotherapy, oxaliplatin, meta-analyses

## Abstract

A meta-analysis was conducted to compare oxaliplatin-based with fluorouracil-based neoadjuvant chemoradiotherapy and adjuvant chemotherapy for locally advanced rectal cancer. MEDLINE, EMBASE and CENTRAL were systematically searched for relevant randomized controlled trials (RCTs) until January 31 2017. Review Manager (version 5.3) was used to analyze the data. Dichotomous data were calculated by odds ratio (OR) with 95% confidence intervals (CI). A total of 8 RCTs with 6103 stage II or III rectal cancer patients were analyzed, including 2887 patients with oxaliplatin+fluorouracil regimen and 3216 patients with fluorouracil alone regimen. Compared with fluorouracil-based regimen group, oxaliplatin-based regimen group attained higher pathologic complete response (OR = 1.29, 95% CI: 1.12−1.49, *P* = 0.0005) and 3-year disease-free survival (OR = 1.15, 95% CI: 0.93−1.42, *P* = 0.21), but suffered greater toxicity (OR = 2.07, 95% CI: 1.52−2.83, *P* < 0.00001). Also, there were no significant differences between two regimens in sphincter-sparing surgery rates (OR = 0.94, 95% CI: 0.83−1.06, *P* = 0.33), 5-year disease-free survival (OR = 1.15, 95% CI: 0.93−1.42, *P* = 0.21) and overall survival (3-year, OR = 1.14, 95% CI: 0.98−1.34, *P* = 0.09; 5-year, OR = 1.06, 95% CI: 0.78−1.44, *P* = 0.70). In conclusion, the benefits of adding oxaliplatin to fluorouracil-based neoadjuvant chemoradiotherapy and adjuvant chemotherapy for locally advanced rectal cancer remains controversial, and cannot be considered a standard approach.

## INTRODUCTION

Rectal cancer is a common and lethal disease. In Europe, 342,137 cases colorectal cancer are diagnosed in 2012 [1], and rectal cancer represents about 27% to 58% of cases [2]. In the United States, approximate 39,910 new cases of rectal cancer are diagnosed in 2017 [3]. Surgical resection is the cornerstone of curative therapy for rectal cancer [4]. However, about 55% of patients with rectal cancer are diagnosed at stage II or III [5], and only patients with early stage rectal cancer can attain a high cure rate by surgery [6]. It is a multidisciplinary approach of treatment for rectal cancer, preoperative and postoperative staging are of crucial importance for patients with stage II or III rectal cancer [7]. Several randomized clinical trials demonstrate that preoperative chemoradiation or short-course radiotherapy improves outcomes in locally advanced rectal cancer [8–11]. Preoperative chemoradiotherapy (or short-course radiotherapy alone) followed by total mesorectal excision surgery have markedly reduced local recurrence rates in stage II or III rectal cancer to well below 10% at 5 years in recent trials [8, 9, 12]. Therefore, this approach is considered one of the standard treatment strategy, especially for locally advanced rectal cancer. Moreover, postoperative chemotherapy is controversial for rectal cancer patients with preoperative chemoradiotherapy and surgery. The benefit of postoperative chemotherapy in rectal cancer patients undergoing preoperative chemoradiotherapy is uncertainty [13–16], although most oncologists recommend it and the majority of patients receive it in United States [17]. Actually, the use of preoperative chemoradiotherapy and postoperative chemotherapy varies among different treatment centers [18, 19]. It is time to look for more-effective systemic treatments.

Fluorouracil-based regimen is considered as a standard approach during neoadjuvant chemoradiotherapy and adjuvant chemotherapy in stage II or III rectal cancer [11, 20, 21]. Fluorouracil-based regimen mainly includes infusions of fluorouracil/leucovorin and oral daily capecitabine [11]. But, fluorouracil-based chemoradiotherapy has no impact on distant metastasis that remain in the 30% range [22]. Oxaliplatin has been adopted as a standard regimen of adjuvant chemotherapy of stage III colon cancer, and adding oxaliplatin to fluorouracil/leucovorin can improve therapeutic efficacy [23–26]. Nevertheless, the benefit of oxaliplatin-based neoadjuvant chemoradiotherapy and adjuvant chemotherapy in stage II or III rectal cancer remains unclear. There are at least eight randomized trials [27–36] investigate the effect of oxaliplatin-based neoadjuvant chemoradiotherapy and adjuvant chemotherapy for stage II or III rectal cancer. However, the efficacy data are controversial. All trials demonstrated that toxicity is clearly worse compared with chemoradiotherapy with a fluoropyrimidine alone and that efficacy is not yet proven. The question remains: if the addition of oxaliplatin in standard neoadjuvant chemoradiotherapy and adjuvant chemotherapy treatment regimen in stage II or III rectal cancer can provide a better survival although with higher toxicity.

There is no consensus on whether the addition of oxaliplatin in neoadjuvant chemoradiotherapy and adjuvant chemotherapy is benefit for patients with stage II or III rectal cancer. The article by Rödel et al. [33] in Lancet Oncology described the results of the latest German study in 2015. A systematic overview by Bujko et al. [19] in 2015 assessed this issue. However, not all eight trials more details were included in that meta-analysis. Hence, we conducted a more updated and better systematic review and meta-analysis on this controversial issue.

## RESULTS

### Study selection

**Figure 1:**
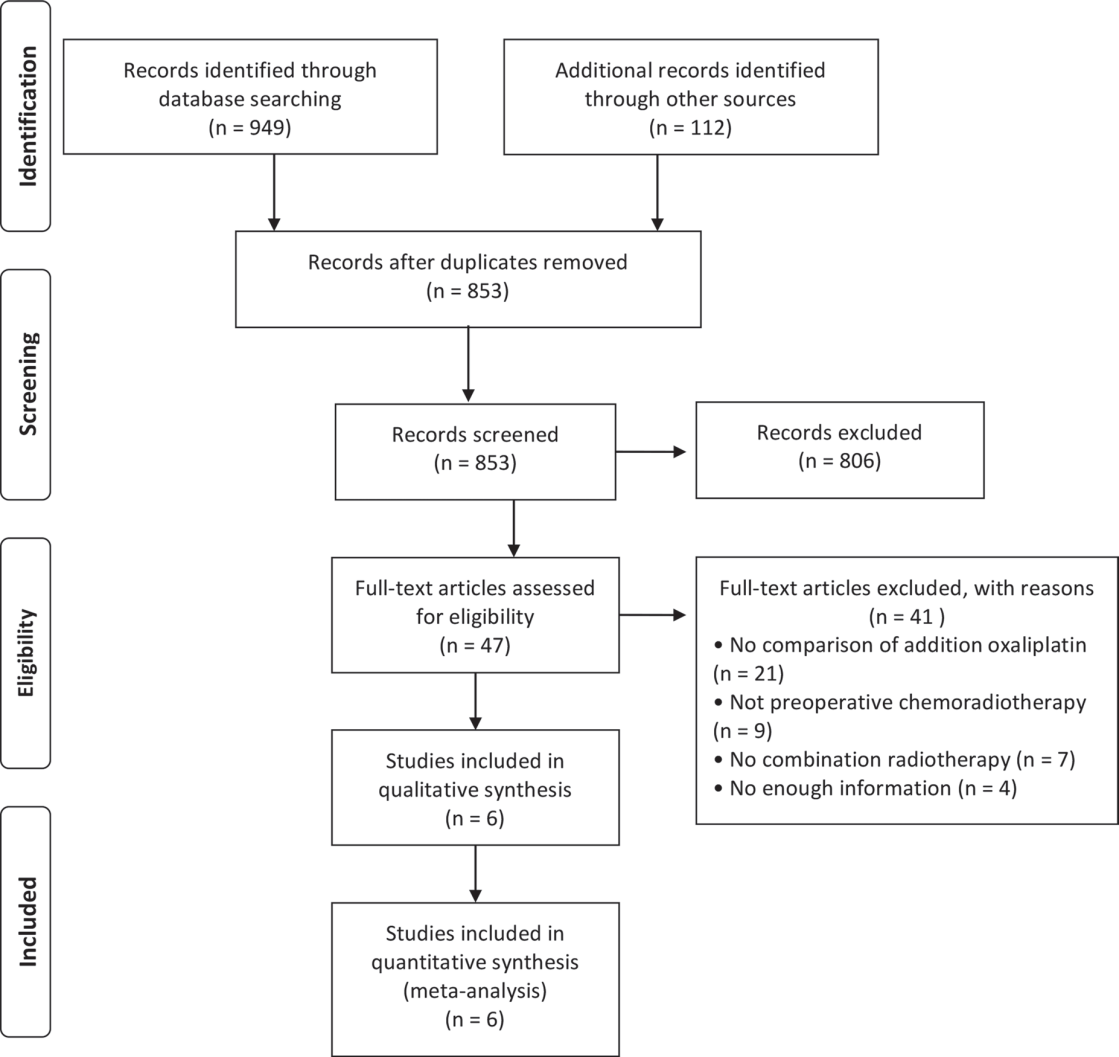
Flow diagram showing the selection of studies for inclusion in this meta-analysis

A total of 853 potential abstracts were identified after deleting out duplication in extensive literature search of electronic database and manual approach until January 31 2017. 804 articles were further ruled out after scanning the title/abstract according to the inclusion and exclusion criteria of this meta-analysis. As for full-text of the remaining 49 articles were subjected to identify. Furthermore, 41 additional articles were ruled out for the reasons described in Figure 1. Finally, 8 RCTs published between 2011 and 2016 were included in quantitative synthesis in this meta-analysis. Figure 1 demonstrates a flow diagram of the detailed selection process.

### Study quality assessment

**Figure 2:**
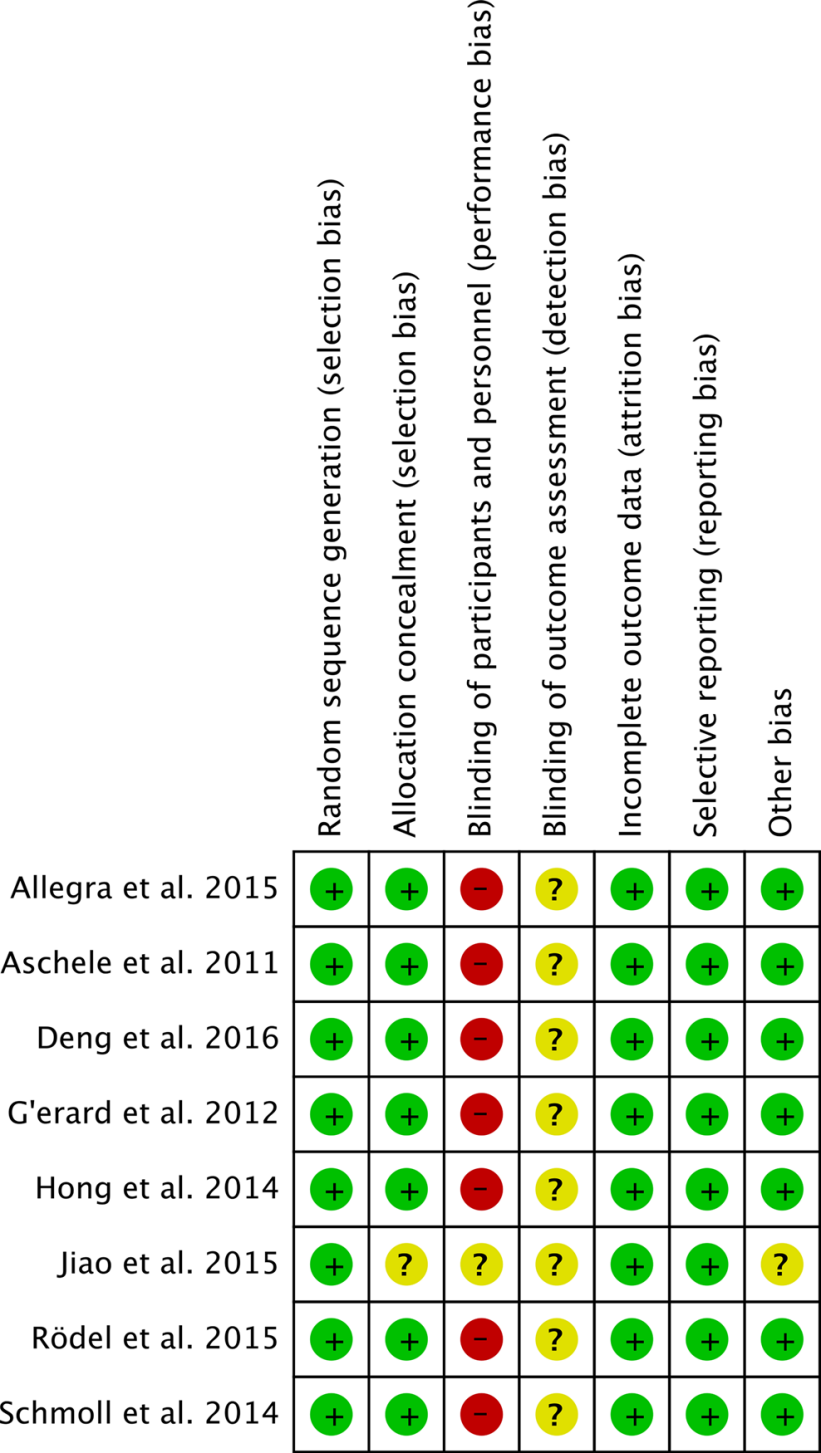
Summary of risk of bias for each selected study assessed by cochrane collaboration's tool.

**Figure 3:**
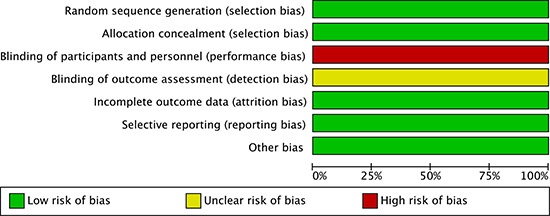
Risk of bias graph about each risk of bias item presented as percentages across all selected studies

Risk of bias assessment was adhered to Cochrane Collaboration's tool, because the included 8 studies were all randomized comparative studies. Figure 2 showed the risk of bias for each study (Figure 2). In all of 8 studies, sequence generation and allocation concealment were randomized, incomplete outcome data and selective reporting were low risk of bias, but healthcare provider and participants were not blinded (Figure 3). Risk of bias of outcome assessment was unclear, due to the outcome data gatherers were not blinded (Figure 3). Accordingly, risk of bias of the included studies in this meta-analysis was low.

### Study characteristics

This meta-analysis included 8 RCTs [27–36] that were conducted in Germany (2), US (1), France (1), Korea (1), Italy (1), and China (2). The sample size of all studies were greater than 200 participants. Overview of the 8 included studies of this meta-analysis was shown in Table 1. This meta-analysis enrolled 6103 patients with stage II/III rectal cancer, including 2887 patients with oxaliplatin-based regimen and 3216 patients with fluorouracil-based regimen. All participants were consecutively enrolled in the statement of studies. Baseline characteristics of these studies were summarized in Table 2.

**Table 1:** Overview of the included randomized control trials of this meta-analysis

Studies(Author, year, country)	Trials	Design	Chemotherapy regimens		Radiation	Follow Up(median)
			Treatment Group	Control Group		
Allegra *et al*. [31, 32],2015, US	NSABP R-04	Multicenter, Open-Label, Randomized, Phase III	Preoperative: OX+FU/CAPEPostoperative: Not specified	Preoperative: FU/CAPEPostoperative: Not specified	45Gy	/
Aschele *et al* [27].,2011, Italy	STAR-01	Multicenter, Open-Label, Randomized, Phase III	Preoperative: OX+FUPostoperative: FU	Preoperative: FUPostoperative: FU	50.4Gy	105.6 m
Deng *et al*. [34],2016, China	FOWARC	Multicenter, Open-Label, Randomized, Phase III	Preoperative: OX+FUPostoperative: OX+FU	Preoperative: FUPostoperative: FU	46-50.4Gy	/
Hong et al. [35],2014, Korea	ADORE	Multicenter, Open-Label, Randomized, Phase II	Preoperative: OX+FUPostoperative: FU	Preoperative: FUPostoperative: FU	NA	38.2 m
G'erard *et al*. [28, 29],2012, France	ACCORD 12/0405	Multicenter, Open-Label, Randomized, Phase III	Preoperative: OX+CAPEPostoperative: FU	Preoperative: CAPEPostoperative: FU	45-50Gy	36.8 m
Jiao *et al*. [36],2015, China	/	Single-center, Open-Label, Randomized	Preoperative: OX+CAPEPostoperative: OX+FU	Preoperative: CAPEPostoperative: OX+FU	50.0Gy	48.7 m
Rödel *et al*. [33],2015, Germany	CAO/ARO/AIO-04	Multicenter, Open-Label, Randomized, Phase III	Preoperative: OX+FUPostoperative: OX+FU	Preoperative: FUPostoperative: FU	50.4Gy	50 m
Schmoll *et al*. [30],2014, Germany	PETACC-6	Multicenter, Open-Label, Randomized, Phase III	Preoperative: OX+CAPEPostoperative: OX+CAPE	Preoperative: CAPEPostoperative: CAPE	45Gy	31 m

Abbreviations: OX, oxaliplatin; FU, fluorouracil; CAPE, capecitabine; m, month; STAR, Studio Terapia Adiuvante Retto; NSABP, National Surgical Adjuvant Breast and Bowel Project; FOWARC, FOLFOX6 Chemotherapy With or Without Radiation in Rectal Cancer; ACCORD, Actions Concertées dans les Cancers Colorectaux et Digestifs; CAO/ARO, Working Group of Surgical Oncology/Working Group of Radiation Oncology.

**Table 2:** Baseline characteristics of the included studies of the meta-analysis

Studies	Arm	No. of patients	Age (years)	Sex (M/F)	Clinical T Category(T2/T3/T4)	Clinical N Category(N0/N1-2)	Clinical Stage (II/III)	Location From Anal Verge (0-5/5-10/>10 cm)
Allegra *et al*. [31, 32], 2015	TreatmentControl	659949	255 (≥ 60)414 (≥ 60)	454/205641/308	NANA	NANA	406/253547/402	130/149119/141
Aschele *et al*. [27], 2011	TreatmentControl	368379	69 (33–75)63 (20–75)	245/123259/120	17/300/50, 1†7/307/65	122/246134/242, 3	NANA	70/213/76, 9†89/202/81, 7†
Deng *et al*. [34],2016	TreatmentControl	165165	52.2 ± 11.854.0 ± 11.9	114/51103/62	3/106/568/100/57	30/13537/128	30/13537/128	83/75/790/70/5
Hong *et al*. [35], 2014, Korea	TreatmentControl	160161	55 (49–63)54 (47–61)	118/42116/45	24/133/324/131/6	58/10265/96	NANA	48/81/3145/89/27
G'erard *et al*. [28, 29], 2012	TreatmentControl	291293	61 (25–80)63 (34–80)	196/95191/102	21/254/1623/255/15	78/211, 2†85/205, 3†	NANA	184 (0–6 cm), 107(> 6 cm)204 (0–6 cm), 89(> 6 cm)
Jiao *et al*. [36],2015, China	TreatmentControl	103103	55.8 ± 2.560.0 ± 2.3	59/4468/35	2/66/353/61/39	22/8123/80	NANA	24/58/2125/57/21
Rödel *et al*. [33], 2015	TreatmentControl	613623	62 ± 1062 ± 10	434/179440/183	22/549/41, 1†32/537/50, 4†	146/452, 15†159/451, 13†	146/452, 15†159/451, 13†	249/302/55, 7†216/336/64, 7†
Schmoll *et al*. [30], 2014*	TreatmentControl	528543	NANA	NANA	NANA	NANA	NANA	NANA

Abbreviations: M, male; F, female; NA, not available; †, Undetermined/data missing; * A preliminary report and later analysis of the trial were presented at the 2013 and 2014 ASCO meeting, but limited by without full-text published article.

### Synthesis of results

### Disease-free survival

**Figure 4:**
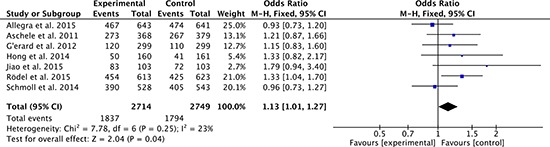
3-year disease-free survival rates of oxaliplatin-based regimen versus fluorouracil-based regimen for stage II or III rectal cancer.

Disease-free survival was the primary endpoint in most studies. There were 7 studies [27–33, 35, 36] comparing 3-year disease-free survival rate between oxaliplatin-based regimen and fluorouracil-based regimen of neoadjuvant chemoradiotherapy and adjuvant chemotherapy for rectal cancer. Heterogeneity was low among the studies (*P* = 0.25, *I*^2^ = 23%), so the fixed effect model was used to pool the outcomes. The result (OR = 1.13; 95% CI = 1.01 to 1.27; *P* = 0.04) indicated that 3-year disease-free survival was significant difference between two groups (Figure 4).

**Figure 5:**
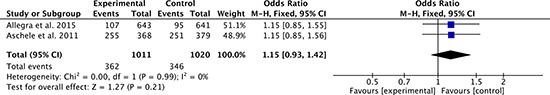
5-year disease-free survival rates of oxaliplatin-based regimen versus fluorouracil-based regimen for stage II or III rectal cancer

In addition, there were 2 trials [27, 31] comparing 5-year disease-free survival rate between two groups. Heterogeneity was none among the studies (*P* = 0.99, *I*^2^ = 0%), so the fixed effect model was used to pool the outcomes. The result (OR = 1.15; 95% CI = 0.93 to 1.42; *P* = 0.21) inferred that 5-year disease-free survival was no significant difference between two groups (Figure 5).

### Overall survival

**Figure 6:**
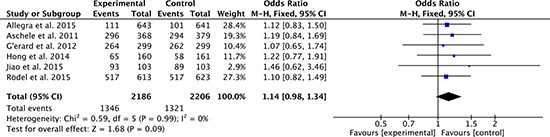
3-year overall survival rates of oxaliplatin-based regimen versus fluorouracil-based regimen for stage II or III rectal cancer

There were 6 studies [27, 28, 31, 33, 35, 36] compared 3-year overall survival rate between oxaliplatin-based regimen group and fluorouracil-based regimen group. Heterogeneity was none among the studies (*P* = 0.99, *I*^2^ = 0%), so the fixed effect model was used to pool the outcomes. The result (OR = 1.14; 95% CI = 0.98 to 1.34; *P* = 0.09) suggested that 3-year overall survival was no significant difference between two groups (Figure 6).

**Figure 7:**
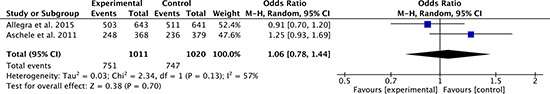
5-year overall survival rates of oxaliplatin-based regimen versus fluorouracil-based regimen for stage II or III rectal cancer

Moreover, 2 studies [27, 31] compared the 5-year overall survival rate between two groups. Heterogeneity was moderate among the studies (*P* = 0.13, *I*^2^ = 57%), so the random effect model was used to pool the outcomes. The result (OR = 1.06; 95% CI = 0.78 to 1.44; *P* = 0.70) implied that 5-year overall survival was no significant difference between two groups (Figure 7).

### Pathologic complete response

**Figure 8:**
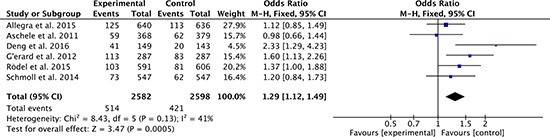
Pathologic complete response of oxaliplatin-based regimen versus fluorouracil-based regimen for stage II or III rectal cancer

Pathologic complete response is an indication of efficacy with regard to oncological outcomes. 6 studies [27–34] compared the pathologic complete response between oxaliplatin-based regimen group and fluorouracil-based regimen group. Heterogeneity was moderate among the studies (*P* = 0.13, *I*^2^ = 41%), so the fixed effect model was used to pool the outcomes. The result (OR = 1.29; 95% CI = 1.12 to 1.49; *P* = 0.0005) indicated that oxaliplatin-based regimen group attained higher pathologic complete response than fluorouracil-based regimen group (Figure 8).

### Overall grade 3–4 toxicities

**Figure 9:**
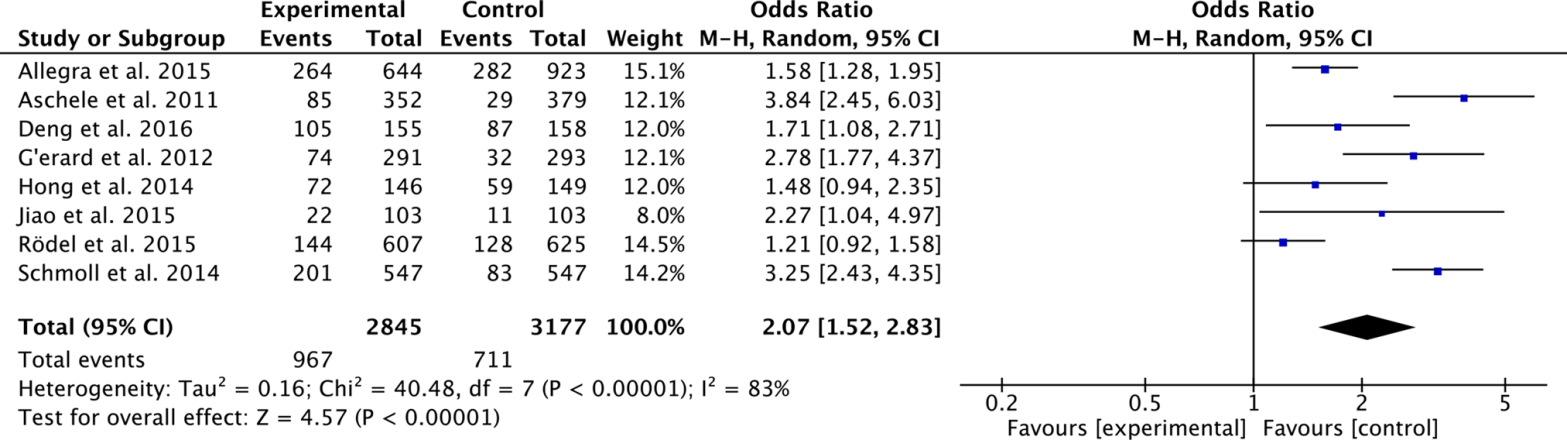
Overall grade 3–4 toxicities of oxaliplatin-based regimen versus fluorouracil-based regimen for stage II or III rectal cancer

There were 8 studies [27–36] compared the overall grade 3–4 toxicities between oxaliplatin-based regimen group and fluorouracil-based regimen group. Heterogeneity was high among the studies (*P* < 0.00001, *I*^2^ = 83%), so the random effect model was used to pool the outcomes. The result (OR = 2.07; 95% CI = 1.52 to 2.83; *P* < 0.00001) suggested that toxicities of oxaliplatin-based regimen group were higher than that of fluorouracil-based regimen group (Figure 9).

### Sphincter-sparing surgery

**Figure 10:**
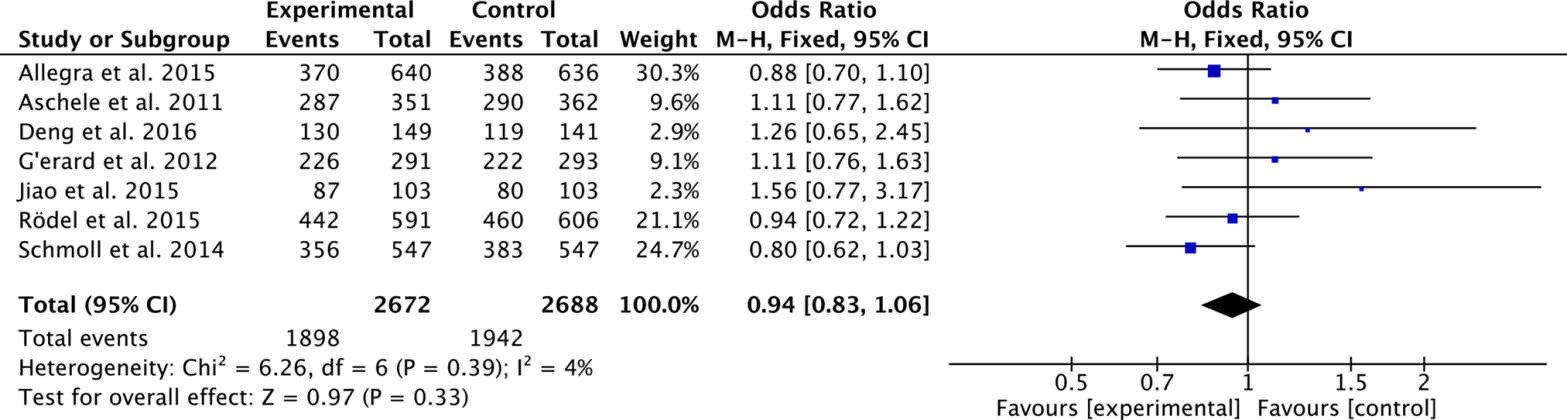
Sphincter-sparing surgery rates of oxaliplatin-based regimen versus fluorouracil-based regimen for stage II or III rectal cancer

There were 7 studies [27–34, 36] compared sphincter-sparing surgery between oxaliplatin-based regimen group and fluorouracil-based regimen group. Heterogeneity was low among the studies (*P* = 0.39, *I*^2^ = 4%), so the fixed effect model was used to pool the outcomes. The result (OR = 0.94; 95% CI = 0.83 to 1.06; *P* = 0.33) inferred that sphincter-sparing surgery was no significant difference between two groups (Figure 10).

### Publication bias

**Figure 11:**
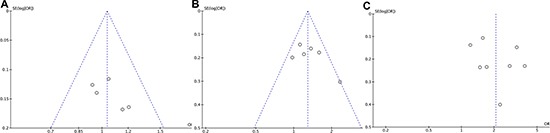
Funnel plots analysis of publication bias (**A**) 3-year disease-free survival (**B**) pathologic complete response (**C**) overall grade 3–4 toxicities.

The funnel plots were utilized to evaluate the publication bias of this meta-analysis. Funnel plot of 3-year disease-free survival (Figure 11A), funnel plot of pathologic complete response (Figure 11B) and funnel plot of overall grade 3–4 toxicities (Figure 11C) were basically inverted and funnel-shaped with bilateral symmetry, indicating that there was no obvious evidence of publication bias.

## DISCUSSION

This meta-analysis compares oxaliplatin-based with fluorouracil-based neoadjuvant chemoradiotherapy and adjuvant chemotherapy for locally advanced rectal cancer. Our pooled results provide convincing evidence for attaining higher pathologic complete response and 3-year disease-free survival, but suffering greater toxicity in oxaliplatin-based regimen. Also, patients with oxaliplatin-based regimen have no improvement in sphincter-sparing surgery, 5-year disease-free survival and overall survival. Upon further analysis, it is no consensus in the real benefit of adding oxaliplatin to neoadjuvant chemoradiotherapy with fluoropyrimidine for locally advanced rectal cancer. On the other hand, patients with locally advanced rectal cancer are likely to benefit from the addition of oxaliplatin to fluoropyrimidine both neoadjuvant and adjuvant treatment. However, the pooled results should be interpreted with caution for the limitations of our study.

Several studies have recently demonstrated that neoadjuvant chemoradiotherapy enhanced the rate of sphincter-sparing surgery and local control for patients with stage II and III rectal cancers [12, 20, 37, 38]. Based on these results, neoadjuvant chemoradiotherapy has become a reference approach for patients with stage II or III rectal cancer. In our study, fluorouracil-based neoadjuvant regimen include two approach: infusion fluorouracil or oral capecitabine. The initial endpoints from NSABP R-04 trial showed that fluorouracil and capecitabine used in rectal cancer resulted in similar rates of pathologic complete response and of sphincter-sparing surgery and surgical downstaging [39]. In addition, the results of NSABP R-04 trial are similar to the recently completed neoadjuvant rectal investigations [12, 20, 37, 38]. These data support composite that our study analyzes infusional fluorouracil and capecitabine together in the neoadjuvant rectal setting. Nevertheless, the 5-FU control regimen was a bolus schedule in two of the larger trials [27, 33], in which the control group used a fluoropyrimidine alone. In the CAO/ARO/AIO-04 study [33], oxaliplatin was given with an entirely different 5-FU schedule and a much less toxic regimen. This weakened the conclusions that adding oxaliplatin increased the overall toxicity. Because the using of a toxic control group (bolus 5-FU schedule) would diminish the ability to see the increase toxicity.

Oxaliplatin has been found to sensitize human cancer cells to the effects of radiation *in vitro*; in addition, several large randomized investigations demonstrate that disease-free survival was significantly enhanced by adding oxaliplatin to 5-fluorouracil in the adjuvant treatment of stage II or III colon cancer [23–26]. While the benefit of adding oxaliplatin to fluorouracil-based chemoradiation in neoadjuvant rectal cancer setting remains unclear.

Pathologic complete response is an indication of efficacy with regard to oncological outcomes. The pooled result confirms that oxaliplatin-based regimen exhibited a signicantly increased pathologic complete response rate (OR = 1.29, 95% CI = 1.12 to 1.49, *P* = 0.0005) than fluorouracil alone. CAO/ARO/AIO-04 [33] and FOWARC [34] trials show that compared with fluorouracil-based agent alone, adding oxaliplatin regimen results higher pathologic complete response, and similar toxicity for patients with stage II or III rectal cancer. In contrast, the other four studies [27–32] reported increased acute toxicity without substantial improvements in pathologic complete response rates. The reasons for this are not completely understood, but might include poorer compliance as a consequence of increased toxic effects, resulting in more dose reduction and treatment interruptions [33].

To our knowledge, disease-free survival in adjuvant colorectal cancer trials and progression-free survival in metastatic cancer are commonly primary endpoints [40]. The results of our meta-analysis confirm that patients with oxaliplatin-based regimen attain higher 3-year disease-free survival, but have no improvement in 5-year disease-free survival and overall survival. Five RCTs included in this meta-analysis have disease-free survival as their primary endpoint. There are four trials, including ACCORD 12/0405-PRODIGE 2 [28, 29], STAR-0 [27], NSABP R-04 [31, 32] and PETACC-6 [30], reported no benefit of 3 or 5 years disease-free survival of oxaliplatin-based; only CAO/ARO/AIO-04 [33] trial reported 3-year disease-free survival improved (75.9% investigational group vs 71.2% control group, *p* = 0.03). However, it must be noted that CAO/ARO/AIO-04 [33] and PETACC-6 [30] added oxaliplatin to both neoadjuvant chemoradiotherapy and adjuvant chemotherapy with fluorouracil-based regimen. Two further RCTs [35, 41] investigated adjuvant fluorouracil-based chemotherapy with oxaliplatin after standard preoperative fluorouracil-based preoperative chemoradiotherapy for stage II or III rectal cancer. CHRONICLE [41] trial closed prematurely because of poor patient accrual. ADORE [35] trial showed improved disease-free survival when oxaliplatin was added to adjuvant chemotherapy after preoperative fluorouracil-based chemoradiotherapy and surgery for stage II or III rectal cancer. Why an increase in the percentage of pathologic complete response do not correspond to an increase in long-term survival indicators (e.g. 5-year disease-free survival and overall survival)? One reason is the selected studies lacked long-term follow-up data for some patients and only 2 studies [27, 31] have an average follow-up time of more than 5 years. Another likely reason is pathologic complete response, as a surrogate index for curative effect, does not completely represent the long-term survival benefit for locally advanced rectal cancer.

Heterogeneity is a classical limitation of meta-analysis, and high heterogeneity may prevent educing convincing conclusions. Moderate to high heterogeneity in this meta-analysis were found only for analyses on pathologic complete response and overall grade 3+ toxicities. Instead, no significant heterogeneity was detected for analyses on disease-free survival, overall survival and sphincter-sparing surgery. All of these increase the accuracy and reliability of the result.

This meta-analysis has several limitations. First, stage II rectal cancer of the 8 selected studies in this meta-analysis is not further divided into low risk and high risk. Today, there is no consensus in some stage II rectal cancer on the real benefit of neoadjuvant chemotherapy (with 5-FU) followed by surgery. Stage II low risk rectal cancers probably do not need a tall chemotherapy with 5-FU or 5-FU plus oxaliplatin. Therefore, the results can be completely different according to this classification schemes. Second, data of long-term outcomes of the selected studies are deficient. Tumor locoregional relapse frequently appears at 4 or 5 years after surgery, when we used neoadjuvant chemoradiotherapy or adjuvant chemotherapy. So, the endpoints should be 5-years disease free survival and complete pathological response with a long-term follow-up. In all the 8 selected studies, only 2 studies [27, 31] show the 5-year disease free survival results and one [33] presents 3-year free survival results. Third, the radiation dose in neoadjuvant therapy as adjuvant therapy schemes, are different in the selected studies of this meta-analysis. Finally, preoperative fluorouracil-based chemotherapy regimen is diverse and the Rodel et al. [33] is the only one that proceed with oxaliplatin plus 5-Fluorouracil in the adjuvant therapy in one of branch of the trial. The ample variety of the chemotherapy schemes may jeopardize the conclusions. Therefore, the results should be interpreted with cautious due to the aforementioned limitations and further large-scale, well-designed RCTs on this topic are still needed.

In conclusion, this meta-analysis compares oxaliplatin plus fluorouracil regimen with fluorouracil alone neoadjuvant chemoradiotherapy and adjuvant chemotherapy for locally advanced rectal cancer. Patients with oxaliplatin-based regimen attain higher pathologic complete response and 3-year disease-free survival, but suffer greater toxicity. Also, sphincter-sparing surgery rates, 5-year disease-free survival and overall survival are no difference between two regimens. But, the results are limited by the aforementioned limitations. Hence, the benefits of adding oxaliplatin to fluorouracil-based neoadjuvant chemoradiotherapy and adjuvant chemotherapy for locally advanced rectal cancer remains controversial, and cannot be considered a standard approach.

## MATERIALS AND METHODS

This meta-analysis was conducted adhering to the PRISMA statement and the Cochrane Handbook for Systematic Reviews of Interventions (Version 5.3) to ensure data quality.

### Data sources and search strategy

MEDLINE (Ovid), EMBASE and CENTRAL were searched comprehensively to identify all relevant clinical trials until January 31 2017. The bibliographies of identified articles were manually searched to identify additional studies. Ongoing clinical trials were also searched by two registers for clinical trials (www.clinicaltrials.gov, www.clinicaltrialsregister.eu).

Search strategy was MeSH terms and free-text terms, as well as variation of root words. Terms were used in different Boolean combinations within each database. The search terms included (“Rectal Neoplasms” or “rectal cancer”) and (“Organoplatinum Compounds” or “oxaliplatin”) and (“Radiotherapy”) and (randomized controlled trial). All potentially eligible articles were retained, and then were examined to determine whether meet the inclusion criteria.

### Study inclusion and exclusion criteria

Studies inclusion criteria in this meta-analysis were list as follow: (i) randomized controlled trials (RCTs); (ii) preoperative chemoradiotherapy with fluorouracil and oxaliplatin versus fluorouracil alone in locally advanced rectal cancer; preoperative chemoradiotherapy with capecitabine and oxaliplatin versus capecitabine alone in locally advanced rectal cancer. Locally advanced rectal cancer was defined as clinical (by transrectal ultrasonography and CT scan or MRI) stage II (T3-4N0) or stage III (T1-4N1-2). (iii) results describing the details of oncological outcomes and survival rate.

Studies exclusion criteria were list as follow: (i) articles without original data, such as abstracts, letters, editorials, expert opinions, case reports and reviews; (ii) studies without reporting clinical outcomes of effectiveness; (iii) studies with a sample size less than 100.

### Data extraction

Data of the included studies were independently extracted by two investigators (X.F. and G.T.). Baseline characteristics included first author, year of publication, country, demographics, study design, number of patients in each arm, clinical trial information, primary endpoint, follow up, clinical disease stage, and location from anal verge. Furthermore, the following data were extracted for meta-analysis: disease-free survival, overall survival, pathologic complete response, overall grade 3–4 toxicities, and sphincter-sparing surgery. Disease-free survival was defined as the time between randomization and any of the following events: death, local relapse or distant metastasis, or second cancer, whichever occurred first. Pathologic complete response was defined as the absence of viable tumor cells in the surgical specimens. Data extracted from the included studies were checked by two other investigators (Z.R. and J.W.) to ensure accuracy and completeness.

### Risk of bias assessment

Risk of bias of the included trials were independently assessed two reviewers (Z.F. and S.L.) according to the Cochrane Collaboration's tool. Good quality criteria studies were as follow: sequence generation randomized; allocation concealment; blinding every participant; complete outcome data; and non-selective outcome reporting. All disagreements were resolved by consensus.

### Statistical analysis

The data were analyzed using Review Manager (Version 5.3 for mac). In this meta-analysis, all variables were dichotomous data, which were calculated by Odds ratio (OR) with 95% confidence intervals (CI). If 95% CI of OR did not include the value 1, *P* < 10.05 was considered to be statistically significant.

Heterogeneity was evaluated by the degree of inconsistency (*I^2^*) and *P* value to assess the variation across studies. If *I^2^* > 50% and *P* < 0.05, a random effect model was used. Otherwise, data were pooled using a fixed effect model. *P* < 0.05 was considered as statistical significance in the integration results. Publication bias was analyzed using a funnel plot for standard error by effect size (log OR).
